# Spatial Pattern and Land Surface Features Associated with Cloud-to-Ground Lightning in Bangladesh: An Exploratory Study

**DOI:** 10.1007/s41748-022-00310-4

**Published:** 2022-05-12

**Authors:** Ashraf Dewan, K. M. Ashraful Islam, Tanzim Rahman Fariha, Md Mahbub Murshed, Asif Ishtiaque, Mohammed Sarfaraz Gani Adnan, Zobaidul Kabir, Mohammad Barad Hossain Chowdhury

**Affiliations:** 1 School of Earth and Planetary Sciences, Curtin University, Kent Street, Bentley, Perth, Western Australia 6102 Australia; 2 Department of Urban and Regional Planning, Chittagong University of Engineering and Technology (CUET), Chattogram, Bangladesh; 3 Department of Meteorology, University of Dhaka, Dhaka, Bangladesh; 4 Department of Geography and Environmental Studies, University of Chittagong, Chittagong, Bangladesh; 5 School for Environment and Sustainability, University of Michigan, Ann Arbor, MI USA; 6 Environmental Change Institute, School of Geography and the Environment, University of Oxford, South Parks Road, Oxford, OX13QY UK; 7 School of Environmental and Life Sciences, University of Newcastle, Newcastle, NSW-2258 Australia; 8 School of Design and Built Environment, Curtin University, Kent Street, Bentley, Perth, Western Australia 6102 Australia

**Keywords:** Cloud-to-ground lightning, Lightning/land index, Topography, Heat flux, Land cover, Irrigation practice, Bangladesh

## Abstract

Severe weather events such as lightning appear to be a significant threat to humans and property in South Asia, an area known for intense convective activity directly related to the tropical climate of these areas. The current study was conducted in Bangladesh and examined the association between cloud-to-ground (CG) lightning and ground surface properties, with the aim of improving existing knowledge regarding this phenomenon. GLD360 data from 2015 to 2020 were used to describe the seasonal lightning climatology. Elevation, land use and land cover, vegetation and surface heat flux data were used to examine all land surface features possibly associated with CG lightning occurrence. Hot and cold spot spatial patterning was calculated using local indicators of spatial association. Results indicated a strong CG lightning seasonality. CG stroke density varied considerably across seasons with the pre-monsoon exhibiting the highest density. This was followed by occurrences in the monsoon season. The March–June period experienced 73% of the total observed. Elevation appeared to influence the post-monsoon CG stroke, however, its role in the other seasons was more difficult to define. The land cover/lightning index indicated that waterbodies and herbaceous wetlands had more influence than other land cover types, both during the day and at night, and it appeared that latent heat flux played a major role. The CG stroke hot and cold spot locations varied diurnally. The findings suggest that large-scale irrigation practices, especially during the pre-monsoon months, can influence the observed spatiotemporal pattern. The production of hotspot maps could be an initial step in the development of a reliable lightning monitoring system and play a part in increasing public awareness of this issue.

**Supplementary Information:**

The online version contains supplementary material available at 10.1007/s41748-022-00310-4.

## Introduction

The Indian subcontinent is known as an active convective region (Zipser et al. 2006) and is one of the most prominent lightning hotspots on earth (Albrecht et al. 2016). The hotspot distribution, however, exhibits spatiotemporal variation due to a variety of factors, including the existence of complex orographic controls (Barros et al. 2004), strong and variable seasonality/diurnality of the convective processes linked to local instability elements (Romatscheke et al. 2010) and the copious moisture supply from the surrounding seas (Chakraborty et al. 2021). Though thunderstorm-related work has been a major research focus for some period in South Asia, a recent surge in lightning studies is evident. This increased interest may be due to a significant rise in reported human fatalities (Yadava et al. 2020; Holle et al. 2019) as well as the availability of long-term flash data from sources such as the lightning imaging sensor (LIS) abroad the Tropical Rainfall Measuring Mission (TRMM). Lightning, particularly cloud-to-ground (CG), is a significant threat to infrastructures and humans, so knowledge of its spatiotemporal pattern can be very useful in developing risk awareness (Vogt and Hodanish 2016), saving lives in an agriculture-based economy (Holle 2016) and assisting in the reduction of impacts on sensitive industries.

Studies that have examined the mechanisms which can trigger lightning events can be broadly divided into two categories. The first category, making up most of the work to date, consists of research that looks at atmospheric instability as a driver for the formation of localised severe storms and lightning (Williams 2005). In this category, meteorological data (e.g., rainfall), thermodynamics (e.g., convective available potential energy, CAPE), dynamic information (e.g., wind shear) and micro-physical parameters such as graupel (Chakraborty et al. 2021; Qie et al. 2020; Lavigne et al. 2019; Mazarakis et al. 2008; Zipser et al. 2006) are examined to identify the spatiotemporal patterns and look at possible uses in forecasting. The second category is relatively new, and hence, not well researched. These studies evaluate the association of lightning with land surface properties (e.g., altitude, slope, soil type, land cover, roughness) (Kar and Liou 2019; Ershova and Punge 2019; Soriano et al. 2019; Aranguren et al. 2014; Vogt and Hodanish 2016; Galanaki et al. 2015; García et al. 2015; Bourscheidt et al. 2009; Kotroni and Lagouvardos 2008; Kilinc and Beringer 2007).

Current climate warming due to widespread urbanisation, industrialisation and the associated land use and land cover (LULC) change, as well as the accompanying anthropogenic forcing, is believed to affect the planetary boundary layer (Soriano et al. 2019; Pielke et al. 2011). A strong heating contrast between differing land cover types can therefore occur. This has significant effects on boundary layer attributes, such as the surface heat flux, which can substantially modify the spatiotemporal pattern of lightning (Sun et al. 2019; Sokol and Rohli 2018; Chate et al. 2017) as lightning is sensitive to temperature variations (Williams 2005). The frequency of lightning incidence may be influenced by the magnitude of this anthropogenic forcing and the intensity of LULC change in a given area (Kar and Liou 2019) as surface properties significantly influence perturbations in surface temperature, and therefore, regional climate variability (Pitman et al. 2011; Rabin et al. 1990). For example, intense irrigation activities are shown to be associated with mesoscale convection and rain patterns in Indian monsoonal regions (Douglas et al. 2009). Similarly, the effect of urbanisation on both the initiation and enhancement of CG lightning is well documented (Kar and Liou 2014; Westcott 1995). A recent study indicated a significant reduction in lightning activity (by 49.16%) in India, attributed to low air pollution resulting from decreased human activity due to the COVID-19 lockdown (Chowdhuri et al. 2020).

Land surface attributes and their relationship with lightning improve our understanding regarding land–atmosphere interactions, however, the results of empirical work show considerable variation. For instance, elevation is shown to influence lightning in Colombia and the Mediterranean region (Aranguren et al. 2014; Kotroni and Lagouvardos 2008) but such a relationship is absent in the Australian and Brazilian cases (Bourscheidt et al. 2009; Kilinc and Beringer 2007). Likewise, the influence of vegetation seems to be dependent on the type (García et al. 2015), roughness (Soriano et al. 2019) and season (Kotroni and Lagouvardos 2008). A positive relationship between CG lightning and LULC is reported in Louisiana, which further shows that latent heat fluctuations have a more direct influence than sensible heat (Sokol and Rohli 2018). Oulkar et al. (2019) noted that the effect of terrain slope and elevation on lightning is stronger in the drier regions of the Indian subcontinent. Station-based days where thunder is recorded are used to explain pre-monsoon lightning activity in Nepal, but the actual locations of maximum lightning stroke do not appear to correspond with areas recording maximum thunder activity (Mäkelä et al. 2014). These studies invariably demonstrate that the link between landscape properties and lightning varies according to the climatic zone (Soriano et al. 2019), the local conditions determining convection (Douglas et al. 2009), and region-specific instability factors (Kellner and Niyogi 2014).

The assessment of spatiotemporal patterning is a powerful tool that can be used to reveal possible elements of geographic phenomenon such as lightning. A variety of techniques are available to obtain statistically significant locations of unusually high or low concentrations (or clusters), commonly referred to as hot and cold spots (Smith et al. 2015). Spatial clustering techniques are commonly used in the study of human geographic issues such as crime (Butt et al. 2020). In recent times, however, these methods have been increasingly used in the analysis of physical geographic phenomena such as floods (Brandt et al. 2020) and urban temperatures (Guerri et al. 2021). Spatial clustering techniques use neighbourhood statistical measures to depict whether features with high (or low) values are clustered (or dispersed) together at a location, so space–time patterning can provide valuable insights into the management of highly localised atmospheric event like lightning. Spatially autocorrelated neighbouring units (i.e., grid or local administrative boundary) tend to share similar environmental conditions that can drive convection (Soriano et al. 2019), so hot and cold spot mapping can be of significant value in building public awareness. Studies in the Indian subcontinent revealed high fatalities among farm labourers who work outside (Yadava et al. 2020) so it can also assist in reducing the vulnerability of the rural populations to a potential lightning strike.

Bangladesh, located on the northeastern flank of the Indian subcontinent, is predominantly lowland and consists of an intricate web of vast water bodies. The ground elevation seldom rises beyond 10 m above sea level (Ohsawa et al. 2000). There are four distinct meteorological seasons: pre-monsoon (March–May), monsoon (June–September), post-monsoon (October–November) and winter (December–February). Rainfall and temperature vary according to the seasons, but 70% of the annual rainfall occurs during the monsoon season. The country is prone to various natural hazards, including local severe storms with associated tornadoes and lightning (Bikos et al. 2016; BBS 2015). Despite pervasive damage caused by flooding and tropical cyclones, deaths associated with a thunderstorm-induced lightning are notable, and perceived to be rising exponentially, particularly during the last few years. Lightning climatology studies, based on LIS data, have revealed two diurnal peaks with a strong seasonality (Dewan et al. 2018a), while CAPE is the single most important factor affecting lightning (Dewan et al. 2018b). Five years (2013–2017) of data from the Global Lightning Dataset (GLD360) is used to determine the association between lightning, agricultural activity, and human fatalities by Holle et al. (2019). This demonstrates a strong correlation between lightning frequency and fatality statistics related to labour-intensive outdoor activities. While the studies mentioned above are useful in depicting lightning distribution and resulting deaths, they do not reveal whether land surface characteristics play a role in determining lightning hotspots, given > 80% of land elevation is < 10 m in Bangladesh. Grid size (Peterson et al. 2021) and the 90-s temporal sampling interval (Holle and Murphy 2017) have been shown to affect satellite-based lightning climatology, so there may be value in developing a finer grid using ground-based datasets to depict localised details of lightning distribution (Albrecht et al. 2016). The majority of the existing studies examining the association between lightning and surface properties are also concentrated on either annual scale (e.g., Sokol and Rohli 2018) or on a single (i.e., warm) season (e.g., Soriano et al. 2019; Vogt and Hodanish 2016).

Yair (2018) demonstrated that land conversion associated with human activities leads to increased surface heterogeneities. As a result, certain biophysical properties of the land surface (including energy balance, roughness and albedo) can be significantly modified (Prijith et al. 2021; Pielke et al. 2011). Consequently, the variability of climatic parameters at local and regional scales is affected by the surface and the overlaying atmosphere (Gentine et al. 2019), causing changes in the timing and location of mesoscale and local-level convection (Pielke et al. 2011; Hanesiak et al. 2004). Bangladesh is slightly smaller than the state of Iowa in America but population-wise it is the tenth-most populous country in the world, with a density of 1115 people/km^2^ (http://www.bbs.gov.bd/). Due to an ever-increasing population and widespread resource exploitation, the country is experiencing extensive LULC change (Xu et al. 2020), with a consequent increase in surface heterogeneity. Land conversion, especially clearing of forests linked with population growth, is thought to be accountable for the ‘perceived’ surge in lightning/high fatalities in recent years in the country. Though lightning incidence and associated deaths fluctuate over time (Holle et al. 2019), Bangladesh’s vulnerability to lightning may increase in future years due to (i) an increase in localised wind shear and CAPE value (two important ingredients accountable for lightning) under warming climate conditions (Glazer et al. 2020); (ii) a growing landscape heterogeneity inducing strong heating contrasts between land surfaces (Soriano et al. 2019; Pielke et al. 2011); and (iii) the interaction of climate with poor socioeconomic and demographic development increasing the overall exposure of the population to adverse impacts.

Existing studies exhibit contrasting results between lightning and surface properties in different geographical regions, so this study is expected to provide valuable insight into understanding the role land surface features have on CG stroke, particularly in a low-lying setting. The aims of this work are to: (a) develop a seasonal CG lightning climatology; (b) reveal spatiotemporal patterning of CG stroke in terms of hot and cold spots; and (c) examine the association between land surface characteristics (e.g., topography and land cover, surface heat flux) and CG stroke.

## Data and Methods

The present study utilises Global Lightning Dataset (GLD360), owned, operated and maintained by Vaisala Oyj (Mallick et al. 2014). The GLD360 networks include very low-frequency sensors (VLF; 500–50 Hz), so they can cover very large distances, i.e., 6000 km (Cohen et al. 2009). An upgrade of the locational algorithm in 2015 improved CG lightning detection efficiency precluding day/night bias (Said and Murphy 2016). Validation work indicated that the median locational accuracy of the dataset is 2–5 km, and the CG detection efficiency is 70% (Pohjola and Mäkelä 2013). Although GLD360 sensors are sensitive to most CG flashes, weaker non-uniform cloud pulses can be interwoven with the stroke data (Rudlosky et al. 2017). For this study, lightning data from 2015 to 2020 were used, with each flash record containing information on date/time, latitudes/longitudes, and peak current polarity. Monthly latent and sensible heat flux data were obtained from https://disc.gsfc.nasa.gov/datasets/FLDAS_NOAH01_C_GL_M_001/summary, Shuttle Radar Topographic Mission (SRTM) v3 digital elevation model (DEM) from https://cmr.earthdata.nasa.gov/search/concepts/C1000000240-LPDAAC_ECS.html, and yearly LULC data from https://lcviewer.vito.be/2015. The original land cover data comprised sixteen categories which were reclassified into seven dominant types for the study. Monthly enhanced vegetation index (EVI) from the 16-day average MODIS13A2 (v6) (https://lpdaac.usgs.gov/products/mod13a2v006/) was also used. Data regarding sunrise and sunset was retrieved from https://www.worlddata.info/asia/bangladesh/sunset.php. A 10 km × 10 km grid size was also adopted to ensure consistency in datasets.

Prior to 11 September 2018, the GLD360 dataset did not separate the CG and IC (intra-cloud) lightning information, so a filtering technique suggested by Nag et al. (2017) is employed in this work. CG strokes with a peak current between − 10 and + 15 kA, are excluded to remove potential cloud pluses. This operation resulted in a total stroke count of 22.72 million out of 50.48 million data points. CG stroke density is computed within a 10 × 10 km grid by adding up individual strokes within a grid and then dividing by the area of the grid. Bangladesh is a relatively small area, so no attempt was made to correct detection efficiency, and strokes that were recorded over the land area during 2015–2020 are aggregated to derive diurnal [according to local standard time (LST)], monthly, seasonal, and annual statistics. To examine the influence of land surface properties (such as elevation) on CG stroke, the cumulative frequency percentage of both land points and the number of CG stroke frequencies are calculated for several elevation bins for the four separate seasons. To understand whether specific LULC categories tend to shape CG stroke, a land cover/lightning index (LLI) proposed by García et al. (2015) is used. The stroke for each LULC category is calculated using LLI. It is then possible to determine the generation of lightning potential by each LULC (Soriano et al. 2019). The unitless LLI is computed at a seasonal scale, with the value > 1 indicating enhanced CG lightning and < 1 denotes suppressed lightning activity over a specified land cover category (García et al. 2015).

To reveal spatial patterns of lightning (as characterised by hot and cold spots), global spatial autocorrelation statistics in terms of Moran’s *I* (a measure of spatial autocorrelation) are employed. This uses seasonal stroke density data. First-order Queen’s contiguity, defined as spatial units sharing a common edge or vertex, is used to conceptualise the spatial adjacency relationship. This denotes the degree of clustering and level of statistical significance among the grids. Local Indicators of Spatial Association (LISA) (Anselin 1995) are used when the results of Moran’s *I* show spatial autocorrelation. The gridded stroke density data is first separated by day and night to account for seasonal variation of day/night length. The rationale of using day and night separately is that lightning deaths tend to vary significantly over time and space (Holle et al. 2019; Dewan et al. 2017). Monte Carlo randomisation of 999 and statistical significance of 0.01 alpha are used to compute the locations of lightning clusters. Statistically significant clusters, denoted by high–high, are referred to as hot spots whilst neighbourhoods (e.g., grids) with low lightning strikes are defined as cold spots (low–low). On the other hand, high–low or low–high clusters are denoted as outliers.

Since the CG stroke density of Bangladesh is affected by a few large lightning days (Holle et al. 2019), the concept of the *thunderstorm hour* has been adopted to eliminate this effect. Using a one-stroke threshold (Huffines and Orville 1999) within the audibility range of 8–20 km of a thunderclap (Changnon 1989), the thunderstorm hour for each day is calculated. A thunderstorm hour is defined by at least one lightning detection (i.e., 1 CG stroke) within a 10 km grid during an hour (DiGangi et al. 2022). Seasonal thunderstorm hourly data are then derived from the monthly values. Monthly heat flux values (in terms of latent and sensible heat) and vegetation data are also prepared for each grid. The role of heat flux and vegetation on lightning is then carried out with a non-parametric Kendall tau correlation. Hourly thunderstorm data and other variables at a monthly scale are employed. To establish the relationship, 18 data points for pre-monsoon (3 months for 6 years), 24 data points for monsoon (4 months for 6 years) and so on for each grid are used.

## Results

### Spatiotemporal Variation

**Fig. 1:**
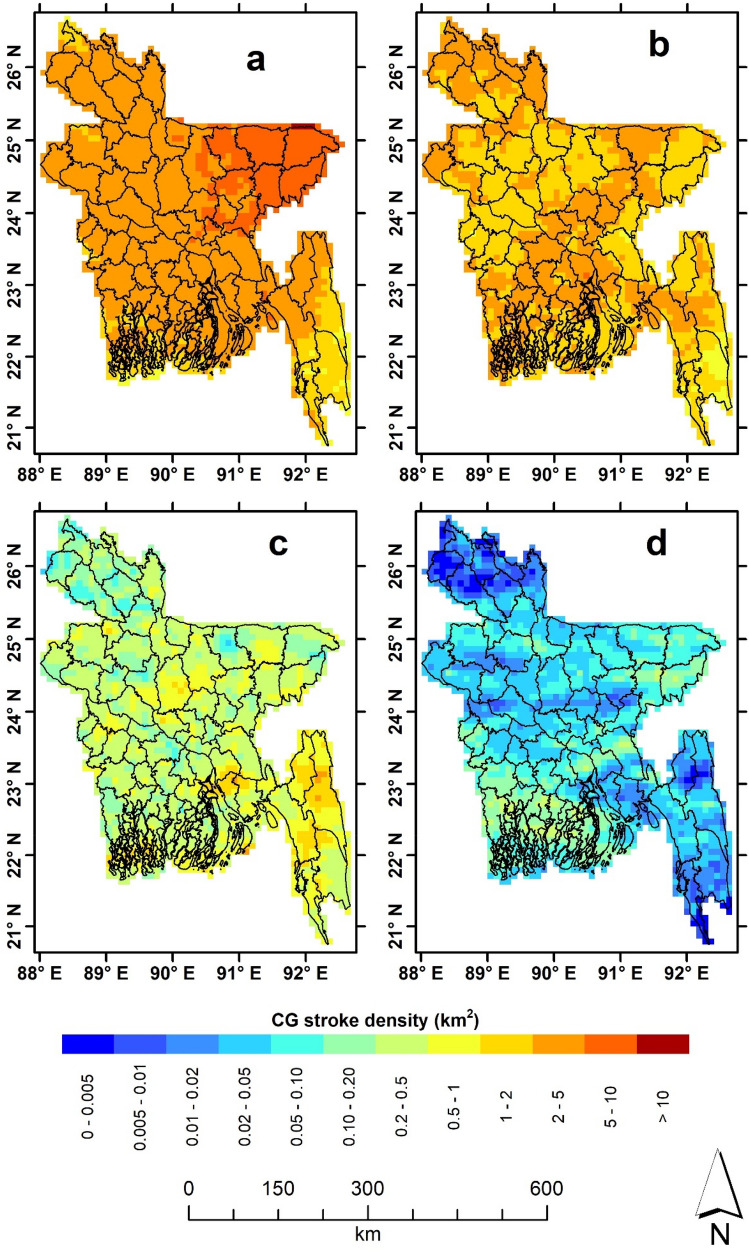
Seasonal CG stroke density over Bangladesh; **a** pre-monsoon; **b** monsoon; **c** post-monsoon and **d** winter. District boundaries are superimposed on gridded CG stroke density

Because of the large spatiotemporal variation in CG stroke quantities, a logarithmic scale is employed to generate the stroke density maps. These are shown in Fig. 1a–d. The analysis, normalised by the length of the season, reveals that lightning density (stroke per km^2^ per season) varies between the seasons, with pre-monsoon (MAM) exhibiting the highest density. Although CG stroke occurs throughout the country during the pre-monsoon months, four eastern district areas (Sunamganj, Sylhet, Habiganj and Maulvibazar) experience high (> 10) CG strokes per km^2^ (Fig. 1a). During the monsoon period (JJAS), lightning density decreases across the country, ranging from 1.0 to 10 strokes per km^2^, with the maximum density mainly be found in Sunamganj (which also recorded the maximum in MAM) and Shariatpur districts. The two eastern districts (Mouvibazar and Sylhet) having maximum CG strokes in MAM (Fig. 1b) also experience less lightning in JJAS. With the end of the monsoon and the start of the post-monsoon (ON) period, the number of strokes reduces significantly, with the density not exceeding 1.0 stroke per km^2^. The districts of Chittagong Hill Tract (CHT), and the central coastal region, exhibit maximum stroke density during ON (Fig. 1c). A substantial reduction in CG stroke density can be seen in the winter season, while the southwest and central coastal districts experience a maximum density of 1.0 stroke per km^2^ (Fig. 1d).

**Fig. 2:**
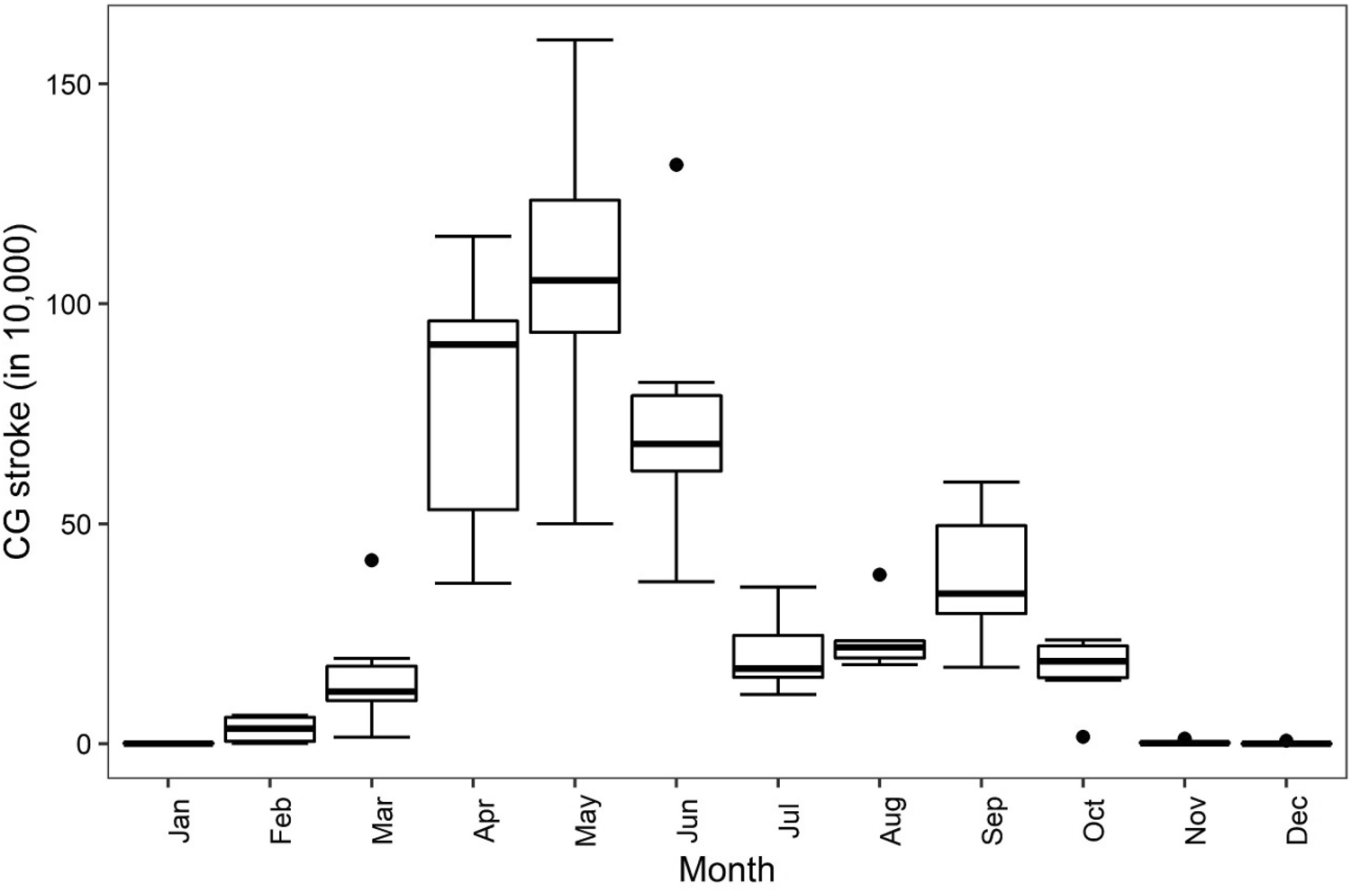
Box-and-whisker plot, showing the monthly distribution of CG stroke. The dots outside the box are outliers. The horizontal bar denotes the median value, and the boxes represent the 25th–75th percentiles

A box-and-whisker plot of the monthly CG stroke (Fig. 2) shows that of 22,718,306 strokes recorded during the 2015–2020 period, the summer month of May records most activity (28.18% of total); this is followed by April (20.78% of total). Among the four monsoon months (JJAS), 19.70% takes place in June and September, comprising 10% of total strokes recorded. Interestingly, 73% of CG lightning occurs between March and June. In contrast, winter months such as December and January experience a significant dip in this activity (Fig. 2).

**Fig. 3:**
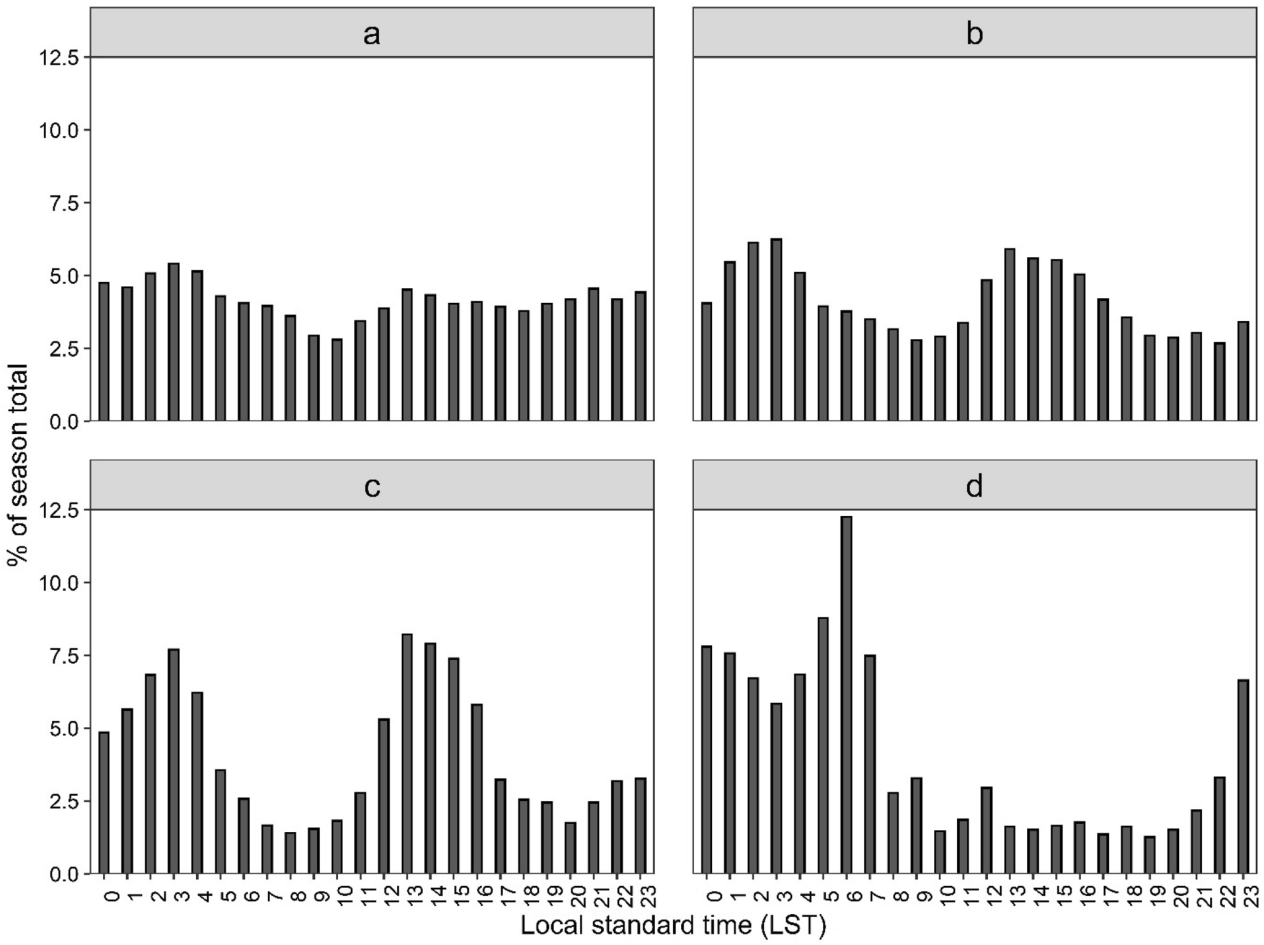
Diurnal CG lightning stroke; **a** pre-monsoon; **b** monsoon; **c** post-monsoon; and **d** winter

Seasonal data (Fig. 3a–d), indicate that for the diurnal CG lightning, strokes occur every hour, with activity higher during the pre-monsoonal and monsoonal months than in the post-monsoon and winter periods. Of the total strokes, 5.41% occur during the early morning (0300 LST) and 2.8% occur at 1000 LST in the pre-monsoon period (Fig. 3a). Intriguingly, maximum lightning activity (6.24%) can also be found during the same time in the monsoon but minimum (2.68%) occurs at 2200 (Fig. 3b). A post-monsoon peak (8.21%) occurs at noon (1300) (Fig. 3c) while winter lightning activity is most frequent (12.25%) at 0600 LST (Fig. 3d). Importantly, the diurnal cycle is highly pronounced during winter, with more nighttime lightning than in the pre-monsoon.

### Effect of Topography and Land Cover

**Fig. 4:**
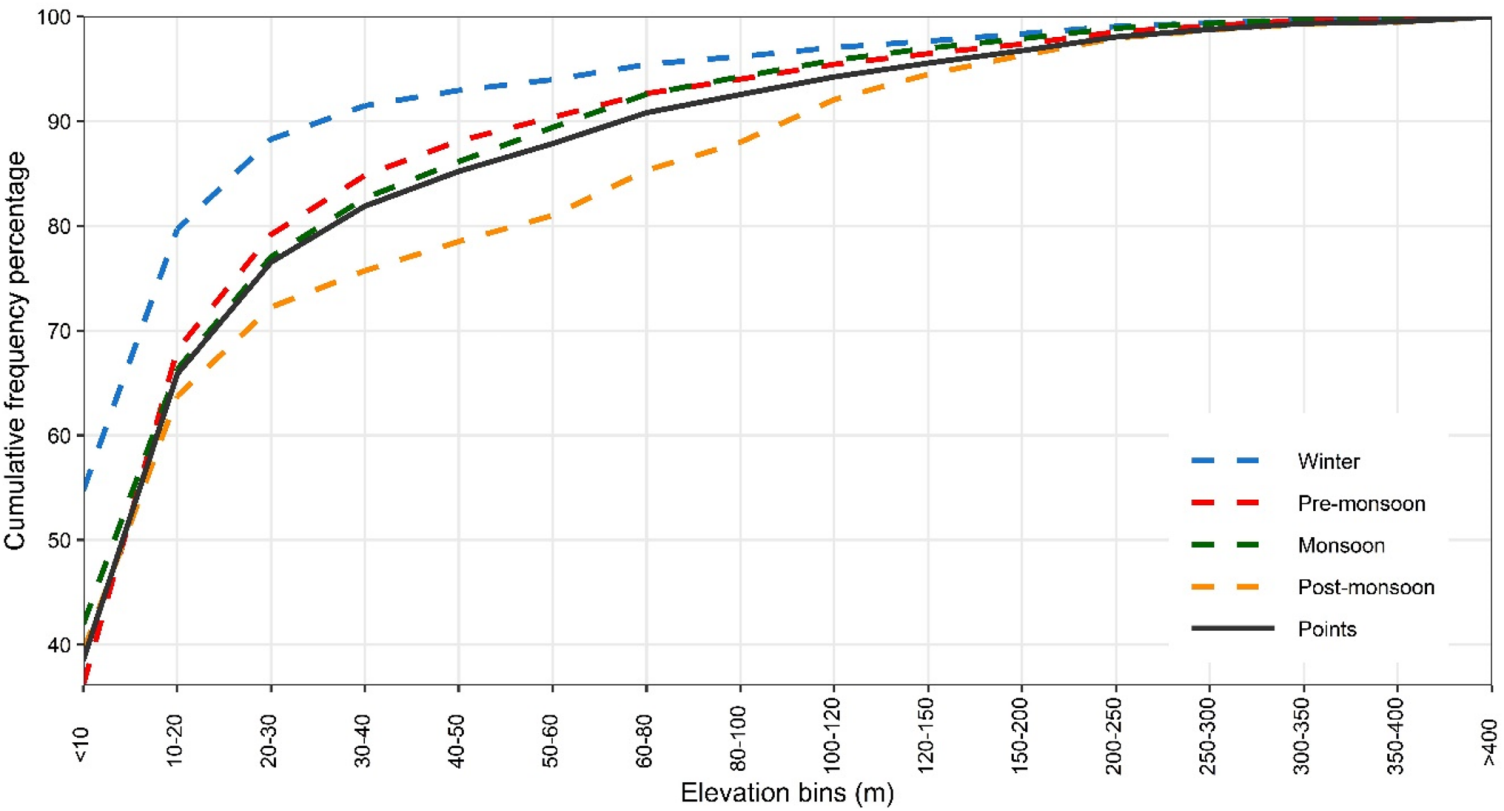
Cumulative percentages of number of land points and lightning activity for various elevation bin across seasons

The effect of elevation on CG stroke is shown in Fig. 4. This plot represents the cumulative percentage of land point number (solid black line) and CG stroke in each season. The analysis underpins that most of the strokes occur over low elevation bins (areas) during the winter season, since the cumulative frequency of CG strokes is well above the cumulative frequency of the land points line (Fig. 4). Specifically, 76.55% land points with < 30 m height experience 88% strokes and 5.76% lands having > 100 m elevation record 2.96% of the lightning during winter. In the post-monsoon, CG stroke appears to be associated with elevation, as the lightning cumulative frequency line is below the land point line. The pre-monsoon and monsoon seasons stroke generally coincide with the cumulative distribution of the land points line, meaning that elevation has little influence on lightning during these two seasons. Results further indicate that 79% of lightning activity occurs within land points of < 30 m, while 4.56% occurs in the elevation bin of > 100 m (during the pre-monsoon). Corresponding lightning values for monsoon is 77% (< 30 elevation) and 4.16% (> 100 m elevation).

**Fig. 5:**
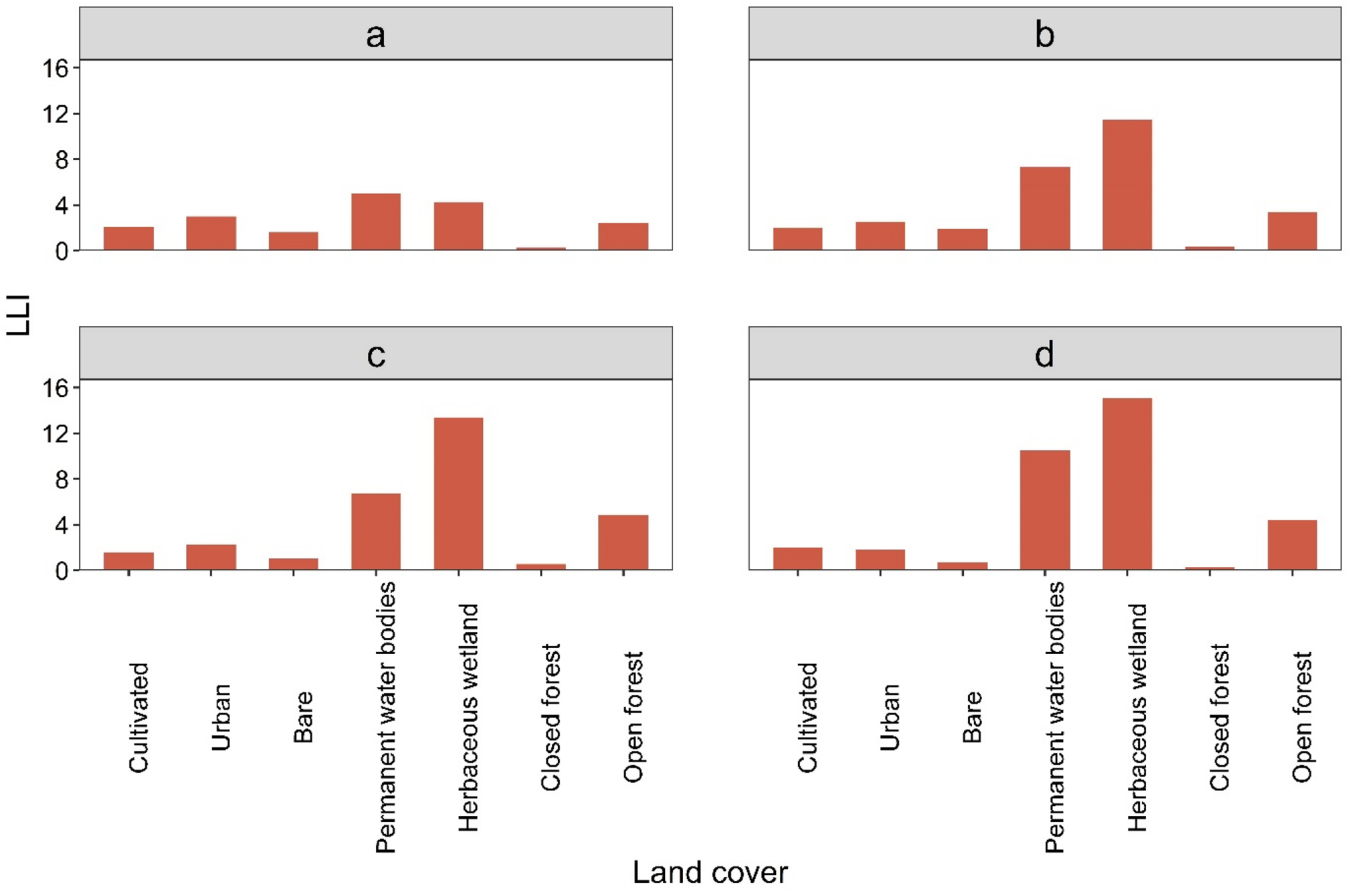
Land cover/lightning index (LLI) during; **a** pre-monsoon; **b** monsoon; **c** post-monsoon; and **d** winter seasons

Figure 5 illustrates LLI for the seven defined land cover categories during the seasons. Waterbody and herbaceous wetland cover exhibit enhanced CG lightning stroke, though the LLI values are relatively low during the pre-monsoon compared with the other seasons. In contrast, closed forest and bare soil categories generally exhibit suppressed CG lightning stroke (Fig. 5). Open forest and urban land covers are also associated with an increased lightning stroke as compared to the cultivated land category. Examination of LLI during the day and at night points to the same outcome, i.e., waterbodies and herbaceous wetland land covers appear to have enhanced CG stroke occurrence relative to other categories in all seasons (supplementary Fig. S1).

### Spatial Pattern of Hotspots

**Fig. 6:**
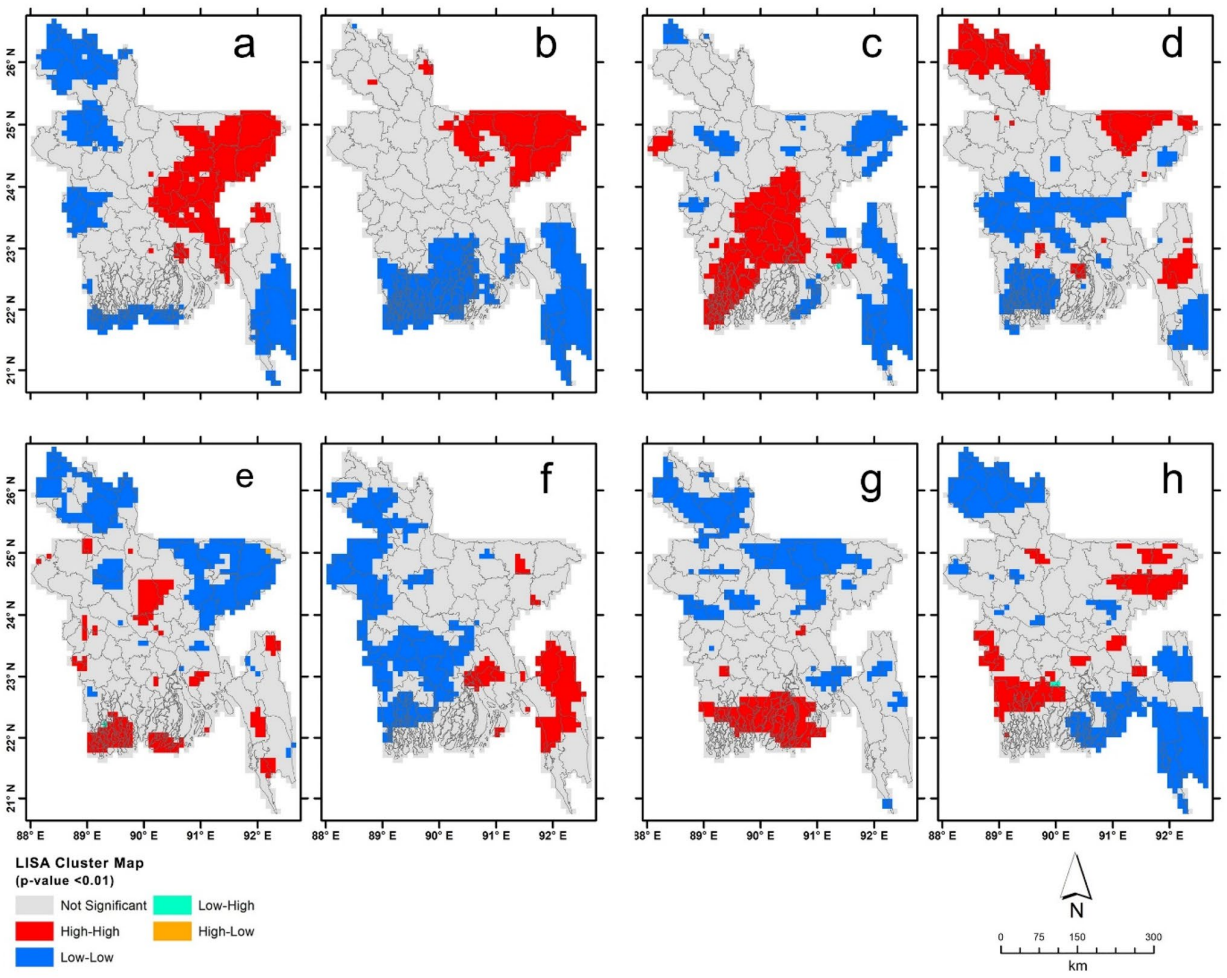
Spatial clusters (hot and cold spots) of CG stroke over Bangladesh: **a** and **b** MAM day and night; **c** and **d** JJAS day and night; **e** and **f** ON day and night; **g** and **h** DJF day and night

The spatial pattern of CG stroke hot and cold spots can be seen in Figs. 6a–h, which indicates that the cluster of CG strokes varies both spatially and temporally, signifying that there is a large variation in the horizontal extent of these spots. For example, multi-centred CG lightning hotspots, denoted by high–high spatial units, can be seen during MAM daytime while a significant reduction in their extent are observed at nighttime (Fig. 6a–b). Conversely multi-centred cold spots, defined by low–low neighbouring units, can generally be observed in the north, northwest, and some coastal districts. During the daytime of JJAS, a large multiple-centred hotspot stretches from the central to the southwest region of the country. Additionally, two single-centred clusters can be seen in Chapai Nawabganj and Feni districts (Fig. 6c). During the monsoon night northern districts such as Thakurgaon, Panchagarh, Niphamari, Lalmonirhat, Kurigram, together with eastern district Sunmganj (centred on 91.43° E and 25.00° N) and southeast district Rangmati (92.25° E and 22.66° N), exhibit lightning hotspots (Fig. 6d). Both single and multi-centred clusters are found around central and coastal districts during ON, while at night a large multi-centred cluster can be observed in the Chittagong Hill Tracts and Chandpur (90.82° E and 23.34° N) along Meghna estuary (Fig. 6e–f). The winter season (DJF) has the least CG lightning activity, and therefore, multi-centred clusters can be found only in southwest and central coastal districts during the daytime (Fig. 6g). Contrarily, lightning hotspots are characterised by both multi- and single-centred around eastern and southwest regions during the nighttime of DJF months (Fig. 6h).

### Factors Influencing CG Lightning

**Fig. 7:**
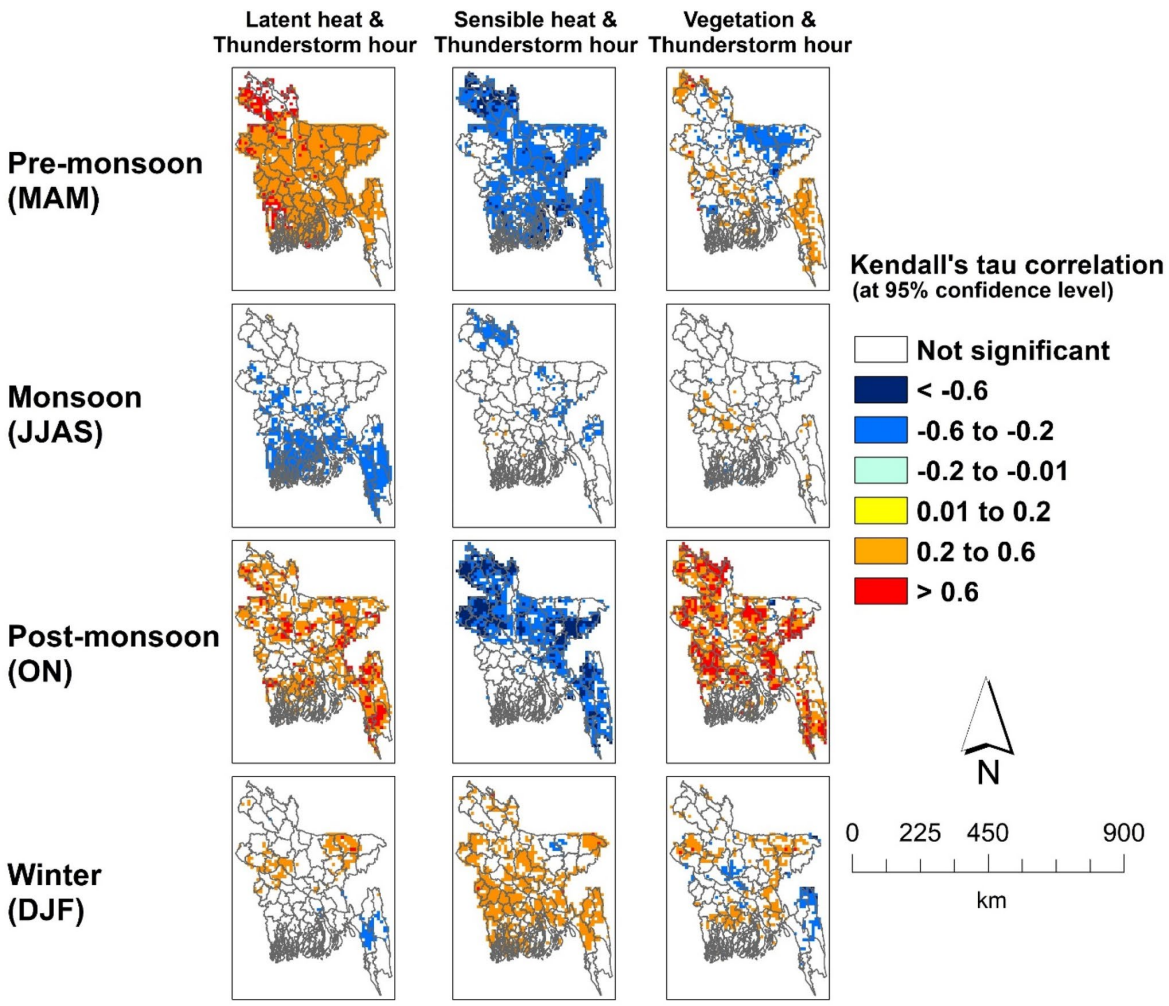
Seasonal spatial correlation maps between thunderstorm hours and latent heat, sensible heat and vegetation

Figure 7 shows seasonal spatial correlation maps, indicating the relationship between thunderstorm hours (obtained from GLD360 data), vegetation and heat flux. During the pre-monsoon, the influence of latent heat flux (*Q*_E_) on the distribution of CG stroke across the country is greater than that of sensible heat flux and vegetation. A strong correlation (*r*^2^ > 0.6 at the 95% CI) can be seen in the grid cells, where irrigation activity is intense during MAM. During monsoon, an association with heat flux variables (sensible and latent heat) is absent, however, vegetation shows a weak correlation for some locations. Moderate to strong correlations can be seen between post-monsoon thunderstorm hours and Q_E_ and vegetation. However, sensible heat flux (*Q*_H_) exhibits a significantly negative correlation in the same season. During the dry months (DJF) of winter, many grids show a statistically significant relationship between *Q*_H_ and thunderstorm hours. Interestingly, the effect of *Q*_E_ diminishes considerably during DJF.

## Discussion

The spatial pattern of CG strokes (Fig. 1) generally agrees with LIS-based climatology, though the distribution does differ during the monsoon and winter seasons (see Fig. 8 of Dewan et al. 2018a). This difference may stem from a range of sources, including the actual grid size used [10 km × 10 km in this work versus 55 km × 55 km in Dewan et al. (2018a)], data used and detection efficiency (Holle and Murphy 2017). Albrecht et al. (2016), for instance, noted that gridding lightning at a fine (higher) resolution is advantageous in depicting localised details in flash density distribution. Temporal patterns (Figs. 2 and 3), on the other hand, generally showed correspondence with the late night–early morning maximum (Dewan et al. 2018a; Nag et al. 2017). Though it is beyond the scope of this work, several factors potentially contribute to the late night–early morning peak seen within Bangladesh. This includes intense convective activity (Islam et al. 2005; Kodama et al. 2005) characterised by a strong diurnality (Romatscheke et al. 2010), peak rainfall events under the influence of nocturnal jet streams (Terao et al. 2006), and terrain-induced local circulations (Kataoka and Satomura 2005; Ohsawa et al. 2001). These phenomena warrant further investigation. The monthly lightning statistics (Fig. 2) may reflect the existence of year-round convective systems in the South Asian region, with the largest occurring between May and October (Qie et al. 2014).

While the literature notes the influence of elevation on lightning in different regions of the world, the current study observed that the impact of elevation on CG strokes can only be seen in the post-monsoon season in Bangladesh (Fig. 4). Although the reason is unclear, empirical studies from Australia (Kilinc and Beringer 2007), Brazil (Bourscheidt et al. 2009) and Mediterranean region (Galanaki et al. 2015) have also observed the same thing. Differential heating of the air over low-lying lands compared with surrounding mountains—analogous to Bangladesh situation—may be responsible for such observation (Bleeker and Andre 1951).

An interesting feature seen, when looking at the lightning/land cover index (LLI), was that CG lightning figures were greatest in herbaceous wetlands and waterbodies areas when compared to other land covers (Fig. 5, supplementary Fig. S1). The evidence for increased lightning activity over urban, cropland and forest land covers is well documented (Soriano et al. 2019; Sokol and Rohli 2018; García et al. 2015), however, the evidence of the role of water bodies in enhancing lightning is less clear-cut (Holle and Murphy 2017; Dissing and Verbyla 2003). A Louisiana study recently proposed that the surface heat flux of water bodies and the aquatic bed may be responsible for enhanced lightning over water surfaces (Sokol and Rohli 2018). This possibility may be relevant to this study as large-scale Bangladeshi water features and wetlands are one of the two reliable sources of moisture for the atmospheric boundary layer. This may act as a convection trigger (Medina et al. 2010) though Holle and Murphy (2017) argue that a waterbody, by itself, does not promote convection. Open forest was another land cover which potentially generates lightning, followed by urban surfaces (Fig. 5). This is in accord with research conducted in other regions (Soriano et al. 2019; García et al. 2015).

Spatial clustering of CG strokes revealed that hotspots shifted diurnally, with the degree of movement dependant on the seasons (Fig. 6). Examining the spatial patterning of hotspots in detail indicated that they are, with few exceptions, largely distributed over low-lying areas surrounded by large waterbodies. Although the physical mechanisms of such patterning are unclear, three factors may be contributing to the noted patterns. Firstly, widespread land surface heterogeneity caused by rapid land-use change in Bangladesh may affect local land–atmospheric interactions. Both observational and modelling studies have indicated that surface heterogeneity can modify regional climatic and environmental factors such as temperature, humidity, wind fields, heat flux and surface roughness at various spatiotemporal scales (Chen et al. 2020; Sun et al. 2019; Ryu et al. 2016; Lawrence and Vandecar 2015; Kellner and Niyogi 2014; Alexander 2011; Pielke et al. 2011). These parameters, either singly or in combination, can influence the dynamic and thermodynamic properties of the air, and, therefore, initiate atmospheric instability (Deng et al. 2014). For instance, deep convection linked with increased asymmetrical surface heating (Williams and Stanfill 2002) and low-level convergence resulting from contrasting land use (Lee and Kimura 2001) can initiate local convection, which in turn, can promote thunderstorm development (Niyogi et al. 2006) and lightning events (Penki and Kamra 2013). Secondly, large-scale irrigation practices, especially in the pre-monsoon season, could affect local climate due to their influence on the regional moisture flux (Adel 2022; Douglas et al. 2009), redistribution of heat flux (Lee et al. 2009), and the land surface albedo and radiation budget (Chen and Dirmeyer 2017; Deng et al. 2014). Since 9.03 million ha of an available 14.4 million ha of land, are cultivable, of which 7.56 million ha are normally irrigated (https://en.banglapedia.org/index.php/Agriculture), intense irrigation activities may result in greater latent heat generation in the plains. Medina et al. (2010) demonstrated that high daytime latent heat flux over waterbodies is linked with a large extent stratiform convective system growth in Bangladesh. Our finding of a statistically significant relationship between thunderstorm hours and latent heat flux during MAM and ON (Fig. 7) may provide evidence that lowland agricultural practices, along with the numerous water bodies present in these areas, may potentially trigger deep convection and enhanced lightning conditions. The difference in latent and sensible heat fluxes caused by land cover transitional zones (very typical of Bangladesh) can also affect boundary layer processes and initiate local convection (Prijith et al. 2021; Kellner and Niyogi 2014). High atmospheric moisture levels resulting from substantial amounts of rainfall occurring in the monsoon season are more likely to influence CG stroke than any latent heat flux effects. Thirdly, convective systems over low-lying areas contain more convective cells than those found over the foothill or plateau regions. This in turn produces more lightning (Xu 2013), as noted in works by Luo et al. (2013) and Hanesiak et al. (2004). Both reported that flat land, and land dedicated to agricultural activities, promote intense convection. This process is likely to be relevant to Bangladesh given its position within the Ganges–Brahmaputra–Meghna (GBM) deltaic plains. Large CAPE (Bikos et al. 2016) and strong wind shear (Glazer et al. 2020) are other factors believed responsible for the strong convection activity observed over the Bangladesh landmass.

This study explored the relationship between elevation, land cover, spatial clustering and CG stroke. There is, however, scope for further improvement. An analysis of potential dynamic/thermodynamic factors initiating local convection is recommended. As noted earlier, the late night–early morning lightning maximum needs further study since most lightning fatalities occur during the morning hours (Dewan et al. 2017). A physical process model that can explicitly represent how different land covers affect the occurrence of lightning could be useful. Work is currently in progress to determine the link between local convection and lightning by grouping data into break and burst periods. The impact of irrigation and cropping patterns on local severe storms is also a very promising area for further research. Irrigation is shown to affect CAPE and temperature (Niyogi et al. 2010; Douglas et al. 2009), so work on how agricultural intensification drives local convection could prove valuable. The current work notes that measures to reduce lightning damage and fatalities must consider seasonality. Lightning hotspot maps can be used as an initial step in the development of early warning systems and to provide information to increase public awareness. This is especially important given sections of the rural population spend a substantial amount of time outdoors involved in agricultural activities, and are, therefore, at risk of a lightning strike (Holle et al. 2019). The results of this study are expected to provide essential information for CG lightning forecasting.

## Conclusions

The study used GLD360 data from 2015 to 2020 to examine the seasonal cloud-to-ground lightning climatology of Bangladesh. The association between land surface properties and CG stroke, over all four seasons, was examined. Results revealed that strong seasonality existed in CG stroke events, although this varied over time and space, with the pre-monsoon season recording the maximum, and winter the minimum, stroke density, respectively. The months of March to June experienced 73% of the total lightning activity, but diurnally the late night–early morning showed enhanced CG lightning stroke events. Examination of a possible relationship between lightning, land cover and elevation indicated that post-monsoon season stroke is dependent on elevation, while the pre-monsoon and monsoon periods had little elevation dependency. The LLI indicated that waterbodies and herbaceous wetlands experienced enhanced lightning activity when compared to other land cover categories.

Spatial clustering of lightning, during both day and night across the four seasons, revealed some interesting features. Although statistically significant multi- and single-centred clusters were identified with the aid of LISA, lightning hotspots appeared to be time-dependent, i.e., clusters tended to drift diurnally. Spatial correlations between thunderstorm hours, vegetation and surface heat flux showed that latent heat flux—an indicator of moisture—had a statistically significant influence on CG lightning during the pre-monsoon season. The winter season, however, exhibited a very weak relationship. As neighbouring spatial units tend to share similar environments, lightning hotspot maps developed in this work could assist in the formulation of public policies and the development of public awareness to prevent fatalities in rural Bangladesh. The findings suggested that local studies are essential in determining all possible factors responsible for initiating local convection given that agricultural intensification may have major implications for future lightning impacts, particularly in the countryside. LULC change should also be included in climate modelling to assist in the prediction of extreme weather events, including lightning.

## Supplementary Information

Below is the link to the electronic supplementary material.

Supplementary file1 (DOCX 226 kb)
