# Supplementary material for: Spatial Pattern and Land Surface Features Associated with Cloud-to-Ground Lightning in Bangladesh: An Exploratory Study
Source: Earth Syst Environ. 2022 May 12;6(2):437–51. doi: 10.1007/s41748-022-00310-4 (PMC9095438; doi:10.1007/s41748-022-00310-4)
Supplement: Supplementary file 1 — Supplementary file1 (DOCX 226 kb) [file 41748_2022_310_MOESM1_ESM.docx]

s
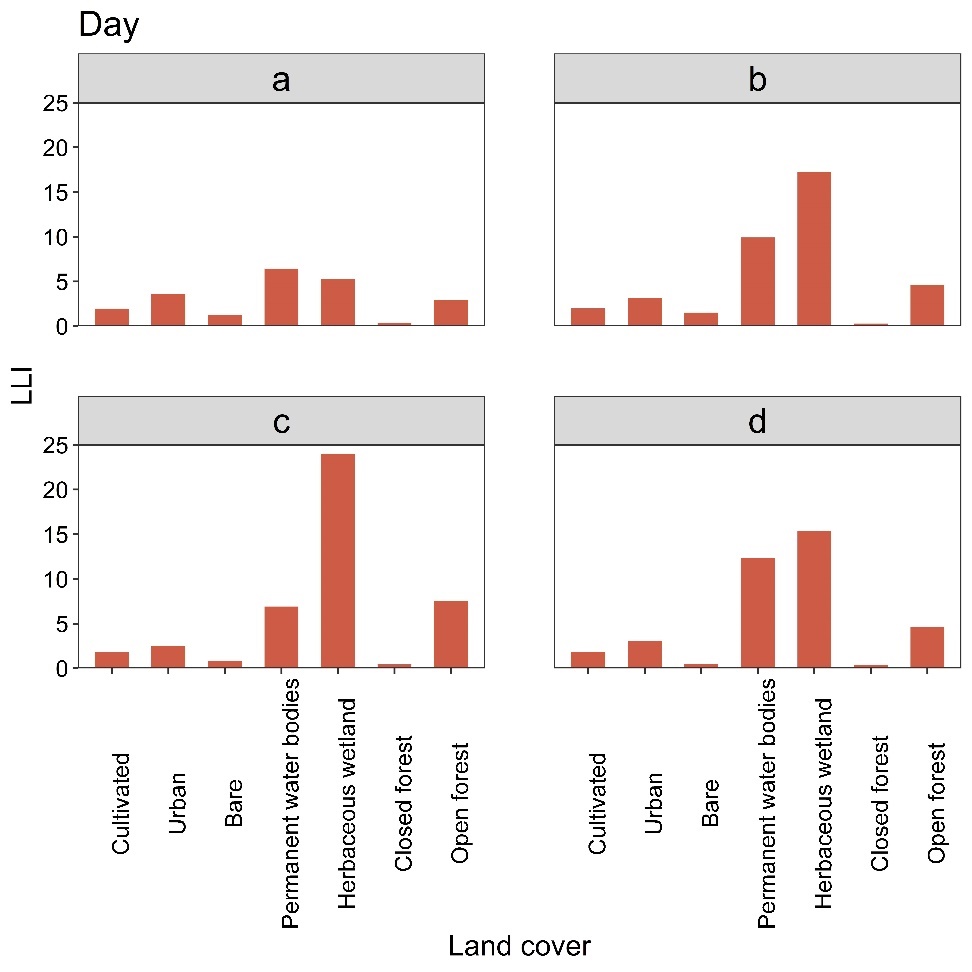


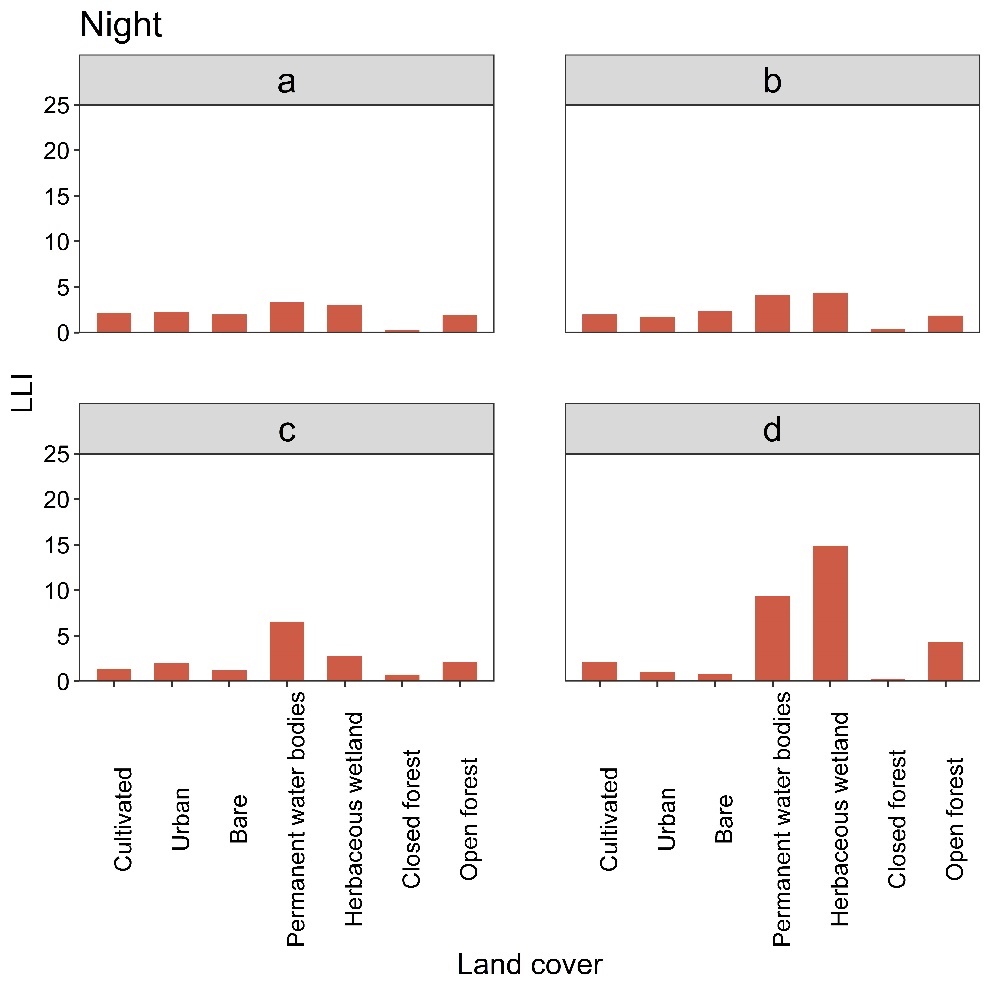


Fig. S1 Seasonal land cover/lightning index (LLI) during day and night: (a) Pre-monsoon; (b) monsoon; (c) post-monsoon; and (d) winter
